# Flexible Asymmetric Supercapacitors with Ultrahigh Energy Density through Synergistic Design of Electrodes

**DOI:** 10.1002/advs.201800784

**Published:** 2018-09-03

**Authors:** Huanan Peng, Guiju Qian, Ning Li, Yao Yao, Tian Lv, Shaokui Cao, Tao Chen

**Affiliations:** ^1^ Shanghai Key Lab of Chemical Assessment and Sustainability School of Chemical Science and Engineering, and Institute of Advanced Study Tongji University Shanghai 200092 P. R. China; ^2^ School of Materials Science and Engineering Zhengzhou University Kexue Road 100 Zhengzhou 450052 P. R. China

**Keywords:** asymmetric, carbon nanotubes, flexible, pseudocapacitive materials, supercapacitors

## Abstract

Despite being among the most researched energy storage devices, supercapacitors have often suffered from their relatively low operating voltage and energy density, which greatly limit their practical applications. In this work, asymmetric supercapacitors (ASCs) are developed by synergistically designing carbon nanotube composite electrodes with 3D porous structures. The resultant ASC devices exhibit an extended operating voltage of 1.8 V, much higher than that of symmetric supercapacitors (≤1.0 V). Significantly, the obtained ASC devices deliver ultrahigh volumetric energy density as high as 19.8 mWh cm^−3^ (corresponding to an areal energy density of 198 µWh cm^−2^), which is the highest value among reported ASC devices. In addition, the ASC devices not only possess outstanding cycling stability and long self‐discharging time, but also exhibit excellent mechanical flexibility under any bending states, even over 5000 bending cycles. The demonstrated flexible ASC devices with high performance are promising to be used as power sources for next‐generation portable and wearable electronics.

Portable, flexible, and wearable electronics^1^, ^2^, ^3^, ^4^, ^5^ (such as displays,^6^ touchscreens,^7^ detectors,^8^ and so on) have attracted increasing attention from academic and industrial fields and represent one of the most promising research areas at present. The performance and practicality of all kinds of electronics greatly depend on the energy supply system. Currently, the most applied energy devices for various electronics are electrochemical energy storage devices containing secondary batteries and supercapacitors. Compared with secondary batteries, supercapacitors not only have higher power densities and longer lifetime and are more eco‐friendly, but also could be easily fabricated into flexible devices with various formats (e.g., fiber‐shaped,^4^, ^5^, ^9^ planar,^10^ and in‐plane^11^) or provided with other functions (e.g., self‐healing,^12^ stretchable,^13^, ^14^, ^15^, ^16^ and transparent^17^) through systematically designing electrodes and electrolytes. Until recently, most of the flexible supercapacitor devices were constructed in a typical symmetric structure with an electrolyte/separator sandwiched between two same type electrodes.^18^, ^19^, ^20^, ^21^ However, the symmetric supercapacitors often suffered from a limited working voltage (≤1.0 V) because of the thermodynamic breakdown potential of water molecules in the used aqueous electrolyte, which resulted in a relatively low energy density, greatly limiting their applications in practice.^22^, ^23^ According to the equation for calculating energy density (*E*) (*E* = 1/2 *CV* ^2^),^24^, ^25^, ^26^, ^27^, ^28^ where *C* is the specific capacitance of supercapacitors and *V* is the operation voltage, it is apparent that the energy density can be greatly enhanced by increasing *V*. Although the working voltage of symmetric supercapacitor devices could be enhanced to more than 2.5 V by using ionic liquid electrolytes, the components in this electrolyte are toxic and not eco‐friendly. In this regard, an alternative approach to achieve higher working voltage is to develop asymmetric supercapacitors (ASCs) by using two different positive and negative electrode materials with well‐separated potential windows. In addition, the energy density of supercapacitors can be improved by increasing their specific capacitance (*C*) through the use of electrochemical double‐layer capacitive materials (e.g., nanocarbon) with pseudocapacitive materials with excellent reversible and rapid redox reactions.^29^, ^30^

For asymmetric supercapacitors, most previous works have mainly focused on exploiting high‐performance positive electrode materials (e.g., MnO_2_,^31^, ^32^ V_2_O_5_,^33^ Ni(OH)_2_,^34^ and conducting polymers^35^), while nanocarbon materials (e.g., graphene^36^ and carbon nanotubes (CNTs)^37^) or their composites with metal oxides or metal nitrides were used as negative electrodes.^17^, ^38^, ^39^, ^40^ As one of the promising layered nanomaterials, molybdenum disulfide (MoS_2_) nanosheets exhibited a high pseudocapacitive property and wide negative potential in neutral electrolyte,^3^, ^30^, ^41^, ^42^, ^43^ but only a small amount of research focused on their application for asymmetric supercapacitors. Both positive and negative electrodes previously reported usually had disordered and free‐standing structures, which resulted in inefficient charge and/or ion transport and thus relatively low performance. In this work, we used CNT network films as the conducting substrates to grow 3D MnO_2_ or MoS_2_ nanosheets on both sides of CNT films in situ. Through this method, the obtained MnO_2_/CNT and MoS_2_/CNT composites had high mass loading (more than 86 wt%) of electrochemically active materials, which was much higher than that of composites fabricated by directly compositing CNTs and MnO_2_ (or MoS_2_), providing the designed electrodes and devices with high pseudocapacitance. Both MnO_2_/CNT and MoS_2_/CNT composites could be used as binder‐free electrodes for supercapacitors. The results, measured by a three‐electrode system in an electrolyte of aqueous lithium chloride (LiCl) solution, showed that MnO_2_/CNT and MoS_2_/CNT composites exhibited high specific capacitance of 1109 and 1296 mF cm^−2^, respectively (corresponding to a gravimetric specific capacitance of 153.9 and 156.6 F g^−1^, respectively), much higher than the results reported previously. The high specific capacitance can be ascribed to the high electrical conductivity of the CNT network and rapid charge (or ion) transport from 3D ordered pseudocapacitive materials to CNT materials. Then, quasi‐solid asymmetric supercapacitors were developed by using aqueous solution of polyvinyl alcohol (PVA) and LiCl as the gel electrolyte, along with MnO_2_/CNT and MoS_2_/CNT composites as the positive and negative electrodes, respectively. By synergistically improving both positive and negative electrodes with fit mass loadings of pseudocapacitive materials to achieve an optimal charge balance, our newly developed asymmetric supercapacitors exhibited high specific capacitance (44 F cm^−3^) and energy density (19.8 mWh cm^−3^), which were significantly higher than the most supercapacitors reported so far. It took over 48.64 h for a charged asymmetric supercapacitor to self‐discharge to one‐half of its initial value (*V* ^1/2^), suggesting excellent charge preservability. Furthermore, these asymmetric supercapacitor devices exhibited outstanding flexibility, and the specific capacitance remained almost unchanged after 5000 cycles of bending.

The positive electrodes of MnO_2_/CNT composites were fabricated by an electrochemical deposition method (see the Experimental Section). Multiwalled CNT network (Figure S1, Supporting Information) film with a thickness of ≈10 µm was used as the conducting substrate. From scanning electron microscope (SEM) images (**Figure** **1**a,b) of the as‐synthesized MnO_2_/CNT composites, it can be clearly seen that uniform MnO_2_ nanosheets with heights of ≈20 µm vertically grow on both sides of the CNT film. The ordered and porous 3D nanostructure of MnO_2_ nanosheets can provide a large electrochemical active surface, efficient electrolyte infiltration, and rapid charge (or ion) transport channels, which may greatly enhance the specific capacitance of electrodes. As shown in Figure 1c, MnO_2_ nanosheets had 13 layers with an interlayer spacing of 0.34 nm. Raman spectra (Figure 1d) showed that the MnO_2_/CNT composite showed typical peaks from both bare CNTs and MnO_2_, which indicated that MnO_2_ composited well with CNTs. The X‐ray diffraction (XRD) patterns (Figure S2, Supporting Information) also showed typical diffraction peaks from CNTs and MnO_2_, once again suggesting MnO_2_ well grown on the CNT film. The appeared broaden and weak intension diffraction peaks near 36° of the MnO_2_/CNT composite indicated the MnO_2_ prepared by the electrochemical deposition contained a large amount of amorphous structures.^44^ Previous research results showed that amorphous MnO_2_ can facilitate the insertion and extraction of protons without structural damage, resulting in high capacitance and stable performance.^31^, ^32^, ^44^ To improve the electrochemical performance of the resulting MnO_2_/CNT composites, the structure and mass loading of MnO_2_ nanosheets on CNT films were systematically adjusted by tuning electrochemical deposition conditions. As shown in Figure S3 (Supporting Information), the density of MnO_2_ nanosheets increased gradually with increasing electrochemical deposition time; as a result of that the mass loading of MnO_2_ increased from 72.3 wt% to 86.3 wt% as the deposition time increased from 10 to 40 min. Please note that it is difficult to realize such a high mass loading of active materials by a simple solution mixing method.

**Figure 1:**
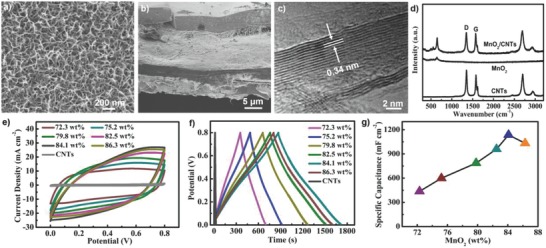
SEM images of MnO_2_/CNT composite from a) top view and b) cross‐sectional view. c) TEM image of MnO_2_ nanosheets in the MnO_2_/CNT composite. d) Raman spectra of bare CNT film, as‐prepared MnO_2_, and MnO_2_/CNT composite film. e) CV curves of MnO_2_/CNT electrodes with different mass loadings of MnO_2_ at a scan rate of 25 mV s^−1^. f) Charge–discharge curves of MnO_2_/CNT electrodes with different mass loadings of MnO_2_ at a current density of 2.0 mA cm^−2^. g) Areal‐specific capacitance of the MnO_2_/CNT electrodes with different contents of MnO_2_ calculated from the GCD curves at a current density of 2.0 mA cm^−2^.

The electrochemical performance of CNT composite electrodes was characterized in a three‐electrode system with an electrochemical working station. For all the CNT composites with different MnO_2_ loading, their cyclic voltammetry (CV) curves (Figure 1e) and galvanostatic charge–discharge (GCD) curves (Figure 1f) appeared in near rectangular and triangular shapes, respectively, showing excellent capacitive behavior. According to the GCD curves, the areal specific capacitance (*C_V_*) of a single electrode can be calculated by using the equation of *C*_S_ = *I*Δ*t*/*S*Δ*V*,^45^ where *I*, Δ*t*, *S*, and Δ*V* represent discharge current, discharge time, the useful area of electrode, and voltage window, respectively. The results (Figure 1g) showed that the areal‐specific capacitance of MnO_2_/CNT composite electrodes increased from 436.4 to 1136 mF cm^−2^ as the mass loading of MnO_2_ increased from 72.3 wt% to 84.1 wt% but then decreased to 1032.0 mF cm^−2^ as the mass loading further increased to 86.3 wt%. The reduction of specific capacitance of MnO_2_/CNT composite electrode with high mass loading can be attributed to the obvious increase of electrical resistance (Figure S4, Supporting Information) of composites as more MnO_2_ nanosheets with poor conductivity were loaded. Therefore, the optimized mass loading of MnO_2_ nanosheets was ≈84.1 wt%. Specially for the MnO_2_/CNT composite containing 84.1 wt% of MnO_2_, its CV curves (Figure S5a, Supporting Information) changed slightly as the scanning rate increased from 10 to 50 mV s^−1^, which revealed that the electrode possessed excellent rate performance. The specific capacitance of the MnO_2_/CNT composite maintained 83.6% as the discharge current density increased from 2.0 to 6.0 mA cm^−2^, which also confirmed that the electrode had good rate performance.

The negative electrodes of MoS_2_/CNT composites were synthesized by the in situ growth of MoS_2_ nanosheets on CNT network films through a typical hydrothermal method. Uniform 3D MoS_2_ nanosheets with a thickness ≈30 µm formed on both sides of the CNT film can be clearly observed from SEM images (**Figure** **2**a,b). As shown in Figure 2c, a layered structure of MoS_2_ nanosheets with interlayer space of 0.28 nm can be seen, which is consistent with previously reported results.^41^ The structure of the MoS_2_ nanosheets was further characterized by Raman spectroscopy (Figure 2d). For pure MoS_2_ nanosheets, two Raman shift peaks at 372 and 404 cm^−1^ corresponded to the in‐plane vibration of Mo and out‐of‐plane vibration of S atoms, which can be ascribed to the E_1g_ and A_1g_ modes of hexagonal MoS_2_ crystals, respectively. The spectrum of the MoS_2_/CNT composite showed Raman shifts derived from both CNTs and MoS_2_, which suggested that MoS_2_ and CNT were successfully composited. The XRD pattern (Figure S6, Supporting Information) of the MoS_2_/CNT composite showed a peak at 2θ = 26.2° derived from the overlap between the (002) plane of CNTs (JCPDS card No. 15‐1621) and the (100) crystal plane of hexagonal MoS_2_ (JCPDS card No. 37‐1492), which also revealed that CNTs and MoS_2_ combined well. The mass loading of MoS_2_ nanosheets in MoS_2_/CNT composites (Figure S7, Supporting Information) can be easily tuned from 77.6 wt% to 89.0 wt% by changing the concentration of precursor. CV and GCD of MoS_2_/CNT composites were further characterized in a three‐electrode system. As shown in Figure 2e,f, CV and GCD curves of the MoS_2_/CNT composites showed near rectangular and triangular shapes, respectively, which revealed that the CNT composite electrodes had an excellent capacitive behavior. In addition, the potential window of MoS_2_/CNT composites ranged from −1 to 0 V, which showed that these MoS_2_/CNT composites could be used as excellent negative electrodes to match with positive MnO_2_/CNT composite electrodes. As expected, the specific capacitance of MoS_2_/CNT composites increased from 499.2 to 1296 mF cm^−2^ as the mass loading of MoS_2_ in CNT composite increased from 77.6 wt% to 87.2 wt%, and then decreased to 1187 mF cm^−2^ as the contents increased to 89.0 wt%. The reduction of specific capacitance at high MoS_2_ loading can be ascribed to the increasing of electrical resistance (Figure S8, Supporting Information) of CNT composites. As Figure S9 (Supporting Information) shows, the CV curves of the MoS_2_/CNT composite electrodes maintained their rectangular shapes well as the scan rate increased from 10 to 50 mV s^−1^, indicating these MoS_2_/CNT composites had excellent rate performance. Especially, GCD curves (Figure S9b, Supporting Information) of the MoS_2_/CNT composites showed perfectly symmetrical shapes under different current densities, exhibiting excellent coulombic efficiency. In addition, the specific capacitance remained over 83.6% as the discharge current density increased from 2.0 to 6.0 mA cm^−2^, showing outstanding rate performance.

**Figure 2:**
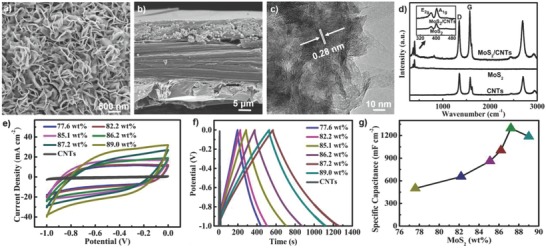
SEM images of MoS_2_/CNT composite from a) top view and b) cross‐sectional view. c) TEM image of MoS_2_ nanosheets in the MoS_2_/CNT composite. d) Raman spectra of bare CNT film, as‐prepared MoS_2_, and MoS_2_/CNT composite film. e) CV curves of MoS_2_/CNT electrodes with different mass loadings of MoS_2_ at a constant scan rate of 25 mV s^−1^. f) Charge–discharge curves of MoS_2_/CNT electrodes with different mass loadings of MoS_2_ at a current density of 2.0 mA cm^−2^. g) Areal‐specific capacitance of the MoS_2_/CNT electrodes with different contents of MoS_2_ calculated from the GCD curves at a current density of 2.0 mA cm^−2^.

The above results confirmed that MnO_2_/CNT and MoS_2_/CNT composites could be used as excellent positive and negative electrodes for ASCs (**Figure** **3**a). To achieve high‐performance ASC devices, it is important to balance the stored charges between the positive and negative electrodes (*q* ^+^ = *q* ^−^). According to the potential windows and specific capacitance of individual positive and negative electrodes in the same electrolyte, the optimal mass loadings of MnO_2_ and MoS_2_ in the corresponding CNT composites was 84.1 wt% and 87.2 wt%, respectively. According to the CV curves (Figure 3b) of MnO_2_/CNT and MoS_2_/CNT electrodes with optimized mass loading, it is expected that the working voltage of the resultant ASC device can be extended to 1.8 V. The ASC devices were fabricated by assembling the positive and negative electrodes together with a gel electrolyte (aqueous solution of PVA and LiCl) used as separator at the same time. CV curves (Figure 3c) of the assembled ASC devices exhibited perfect rectangular shapes with the voltage windows increasing from 0.8 to 1.8 V, indicating excellent capacitive behavior. As shown in Figure 3d, GCD curves of ASC devices within different potential windows (0.8–1.8 V) showed excellent linear slopes and triangular shapes, suggesting ideal capacitive behavior consistent with the CV curves. More importantly, the specific capacitance increased from 332.1 to 420 mF cm^−2^ as the operating potential window extended from 0.8 to 1.8 V (Figure 3e); because of this, the areal energy density of ASC devices dramatically increased from 149.5 to 189 µWh cm^−2^ (corresponding to a volumetric energy density of 18.9 mWh cm^−3^ at 0.186 W cm^−3^). It should be noted that the specific capacitance of the two‐electrode supercapacitor devices was calculated according to the equation of *C*_S_ = *I*Δ*t*/*S*Δ*V*, where *S* is the effective area of the assembled ASC devices. These values are much higher than those of most quasi/all‐solid‐state asymmetric supercapacitors reported previously based on different electrode systems (Figure 3f), such as MnO_2_//Fe_2_O_3_/PPy (0.22 mWh cm^−3^, 165.6 mW cm^−3^),^46^ MnO_2_//Ti‐Fe_2_O_3_@PEDOT (0.89 mWh cm^−3^, 440 mW cm^−3^),^47^ ASC devices with two pieces of carbon cloth grown with hydrogenated single‐crystal ZnO@amorphous ZnO‐doped MnO_2_ core–shell nanocables as electrodes (0.04 mWh cm^−3^, 2.44 mW cm^−3^),^48^ MnO_2_ nanowires//Fe_2_O_3_ nanotubes (0.32 mWh cm^−3^, 139.1 mW cm^−3^),^49^ MnO_2_/graphene//VOS@C (0.87 mWh cm^−3^, 9 mW cm^−3^),^50^ MnO_2_//Fe_2_O_3_ (0.41 mWh cm^−3^, 0.1 W cm^−3^),^51^ and ASC devices from MnO_2_/ZnO core–shell nanorods//specially reduced graphene oxide (0.234 mWh cm^−3^, 0.133 W cm^−3^).^52^ Table S1 (Supporting Information) compares our results with other more previous relevant reports, it can be further found that the energy density of our ASC devices was much higher than those of other reports. The predominant energy density of our newly developed asymmetric supercapacitors can be attributed to the unique 3D porous nanostructures of electrodes and well‐balanced charge storage between positive and negative electrodes. Furthermore, it can be expected the energy densities of ASC devices based on our electrodes can be further enhanced when the electrolyte of ionic liquid or gel with broad working potential windows was used instead of aqueous gel electrolyte.

**Figure 3:**
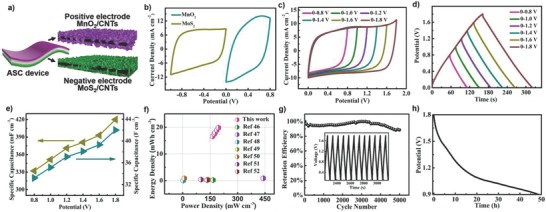
a) Schematic illustration of the flexible ASC. b) CV curves of individual MnO_2_/CNT and MoS_2_/CNT composite electrodes measured in a three‐electrode system at a scan rate of 25 mV s^−1^. c) CV curves of an ASC device at different operating voltages from 0.8 to 1.8 V at a constant scanning rate of 25 mV s^−1^. d) GCD curves of the ASC device collected over different voltages from 0.8 to 1.8 V. e) Areal‐specific capacitance and volumetric‐specific capacitance calculated from the GCD curves obtained at 2.0 mA cm^−2^ as a function of the potential window. f) Comparison of energy density and power density of our ASC device with those of other ASCs reported previously. g) Cycling performance of the ASC device at 5.0 mA cm^−2^ (the inset contains charge–discharge profiles). h) The self‐discharge behavior of an ASC device.

The ASC device also exhibited excellent rate performance, which can be observed from CV curves at different scan rates and GCD curves (Figure S10, Supporting Information) at different charge–discharge currents. In addition, the retention of specific capacitance of our ASC devices remained near 90% of their original values after 5000 charging–discharging cycles (Figure 3g). The excellent cycling retention can be ascribed to that the structures of both positive and negative electrodes maintained well after cycling charge–discharge process (Figure S11, Supporting Information). Self‐discharging performance of our ASC device was also measured (Figure 3h), which showed that the voltage of a charged ASC device self‐discharged to one‐half of the initial value (*V* ^1/2^) in 48.64 h, much longer than that of most supercapacitors reported previously.^53^, ^54^, ^55^, ^56^ The long self‐discharging time of our ASC devices can be attributed to the relatively slow charge distribution across the 3D porous structure and the large specific surface areas of the CNT composites (the specific surface areas of bare CNT film, MnO_2_/CNT composites with mass loading of 75.2 wt% and 84.1 wt% were 176.0, 214.3, and 354.0 m^2^ g^−1^, respectively, as shown in Figure S12, Supporting Information).

To investigate the flexibility of the obtained quasi‐solid‐state ASC devices, the changes of their electrochemical performance were compared under different bending states and numbers of bending cycles. As shown in **Figure** **4**a,b, both CV and GCD curves of the ASC device were perfectly overlapped, almost without change, when the device was bent at any angle and even twisted, exhibiting excellent flexibility. Even after 5000 bending cycles, the electrochemical performance of the ASC device was well maintained (Figure 4c,d), which indicated that our ASC device had excellent mechanical flexibility and stability. The output voltage and current can be easily tuned by connecting multiple devices in series or in parallel (Figure S13, Supporting Information). Figure 4e–h shows the digital photographs of an intelligent watch (operation voltage of 3 V) powered by two ASC devices connected in series. There was almost no change occurring as the ASC devices were bent or folded during usage, showing great potential to be used as power source for practical applications in the fields of flexible electronics.

**Figure 4:**
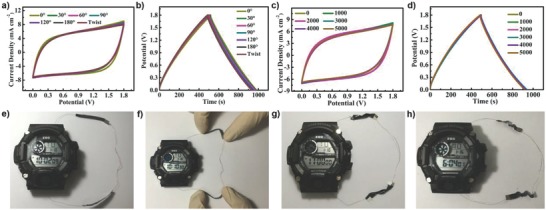
a) CV curves of an ASC device as it was bent at different angles and even twisted at a constant scan rate of 25 mV s^−1^. b) GCD curves of the ASC device when it was bent at different angles and twisted. c) CV curves of the ASC device being bent for different times at a constant scan rate of 25 mV s^−1^. d) GCD curves of the ASC device being bent with different times. e–h) Digital photographs of two ASC devices connected in series to power a watch as the devices were bent under different states.

In conclusion, asymmetric supercapacitors with ultrahigh energy density (19.8 mWh cm^−3^) and excellent mechanical flexibility have been developed by using MnO_2_/CNT composites as the positive electrodes and MoS_2_/CNT composites as the negative electrodes. The 3D porous structures of both CNT composites greatly facilitated the transport of charges and/or ions from pseudocapacitive materials to CNT electrodes, resulting in high specific capacitance. The supercapacitors made in asymmetric structure can efficiently extend their operational voltage, which can in turn result in a higher energy density than that of devices with symmetric structures. Our newly developed ASC devices not only exhibited excellent electrochemical performance but also showed excellent mechanical flexibility and cyclical bending stability. In addition, our ASC devices also exhibited long self‐discharge time, which is important when they are to be used as power sources in practice. These flexible ASC devices with high performance are promising to be used as energy supplies for portable and wearable electronics in the future.

## Experimental Section

*Preparation of MnO_2_/CNT Composites*: CNT network films were purchased from Suzhou (Suzhou Jiedi Nano Science and Technology Co., Ltd.), which were pretreated in concentrated nitric acid for 12 h to remove amorphous carbon and other impurities. The MnO_2_/CNT composites were synthesized through an electrochemical method in a three‐electrode system, where the treated CNT film, a platinum sheet, and Ag/AgCl were used as the working electrode, counter electrode, and reference electrode, respectively. The mixed aqueous solution of 0.05 m MnSO_4_, 0.05 m CH_3_COONa, and 10% ethanol by volume was used as the electrolyte. The electrochemical deposition was conducted at a constant current density of 5.0 mA cm^−2^, and the mass loading of MnO_2_ in CNT composites was adjusted by changing the deposition time. The as‐prepared MnO_2_/CNT composites were rinsed with deionized water several times, followed by drying in a vacuum oven at 60 °C for 2 h.

*Preparation of MoS_2_/CNT Composites*: MoS_2_/CNT composites were fabricated through a hydrothermal method.^57^ The detailed steps were as follows: 1) 3.64 g (65.4 mmol L^−1^) (NH_4_)_6_Mo_7_O_24_ ∙ 4H_2_O and 3.20 g (0.93 mol L^−1^) CH_4_N_2_S were dissolved in 45 mL deionized water, and then magnetically stirred for 1 h to form a homogeneous solution; 2) The obtained uniform mixed solution was transferred to a 25 mL polytetrafluoroethylene (PTFE)‐lined high pressure reactor, and the treated CNT films were immersed in the above solution for 9 h at a temperature of 200 °C for the reaction to occur; 3) After the reactor cooled down to room temperature, the as‐prepared MoS_2_/CNT composites were alternately rinsed with deionized water and alcohol several times, and then, after drying in a vacuum oven at 60 °C overnight, the MoS_2_/CNT composite electrodes were obtained. The contents of MoS_2_ in CNT composites were tuned by changing the concentration of (NH_4_) _6_Mo_7_O_24_ ∙ 4H_2_O from 9.33 mol L^−1^ to 84.1 mmol L^−1^ and CH_4_N_2_S from 0.13 to 1.2 mol L^−1^ in the precursor. The mass loading for both MnO_2_/CNT and MoS_2_/CNT composites was weighed before and after growing MnO_2_ (or MoS_2_) by using an electronic analytical balance.

*Assembly of Asymmetric Supercapacitors*: PVA/LiCl gel solution was used as the electrolyte, which was prepared by adding 1.0 g LiCl∙H_2_O and 1.0 g PVA into 10 mL deionized water, and stirring vigorously at 85 °C for 2 h until the solution became clear. To achieve good interfacial contact between electrodes and electrolyte, a vacuum system was used to infiltrate the electrolyte solution into the CNT composite positive and negative electrodes. The electrolyte‐infiltrated and electrolyte‐coated CNT composite electrodes were then transferred into a vacuum oven and dried at 60 °C for 1 h to remove water from the electrolyte solution. The ASC devices were assembled by pressing the electrolyte‐coated positive and negative electrodes together.

*Characterization and Electrochemical Performance Measurements*: The morphology and structure of CNT composites were characterized by high‐resolution field emission scanning electron microscopy (Hitachi S‐4800), high‐resolution transmission electron microscopy (JEOL‐2010), and XRD spectroscopy (D8 Advance, Bruker) with Cu Kα radiation (*V* = 30 kV, *I* = 25 mA). Raman spectra were recorded on a Renishaw Raman spectrometer equipped with a 514 nm laser. The electrochemical performances of electrodes and ASC devices were evaluated by cyclic voltammetry and galvanostatic charge–discharge on an electrochemical workstation (CHI 760E, Shanghai Chenhua). The electrochemical performance of the positive and negative electrodes was measured in a three‐electrode configuration using LiCl aqueous solution (1 mol L^−1^) as the electrolyte. The fabricated electrode materials were directly used as the working electrodes, and a Pt plate and Ag/AgCl were used as the counter and reference electrodes, respectively.

## Conflict of Interest

The authors declare no conflict of interest.

## Supporting information

Supplementary
