# Supplementary material for: Flexible Asymmetric Supercapacitors with Ultrahigh Energy Density through Synergistic Design of Electrodes
Source: Adv Sci (Weinh). 2018 Sep 3;5(11):1800784. doi: 10.1002/advs.201800784 (PMC6247045; doi:10.1002/advs.201800784)
Supplement: Supplementary file 1 — Supplementary [file ADVS-5-1800784-s001.pdf]

Copyright WILEY-VCH Verlag GmbH & Co. KGaA, 69469 Weinheim, Germany, 2018.

ADVANCED SCIENCE
Open Access

Supporting Information

for *Adv. Sci.,* DOI: 10.1002/advs.201800784

Flexible Asymmetric Supercapacitors with Ultrahigh Energy Density through Synergistic Design of Electrodes

Huanan Peng, Guiju Qian, Ning Li, Yao Yao, Tian Lv, Shaokui Cao, and Tao Chen**

Supporting Information

Flexible Asymmetric Supercapacitors with Ultrahigh Energy Density through Synergistic Design of Electrodes

Huanan Peng, Guiju Qian, Ning Li, Yao Yao, Tian Lv, Shaokui Cao, and Tao Chen

G. Qian, N. Li, Y. Yao, Dr. T. Lv, Prof. T. Chen

Shanghai Key Lab of Chemical Assessment and Sustainability, School of Chemical Science and Engineering, and Institute of Advanced Study, Tongji University, Shanghai, 200092, PR China

E–mail: tchen@tongji.edu.cn

H. Peng, Prof. S. Cao

School of Materials Science and Engineering, Zhengzhou University, Kexue Road 100, Zhengzhou, 450052, PR China

E–mail: caoshaokui@zzu.edu.cn

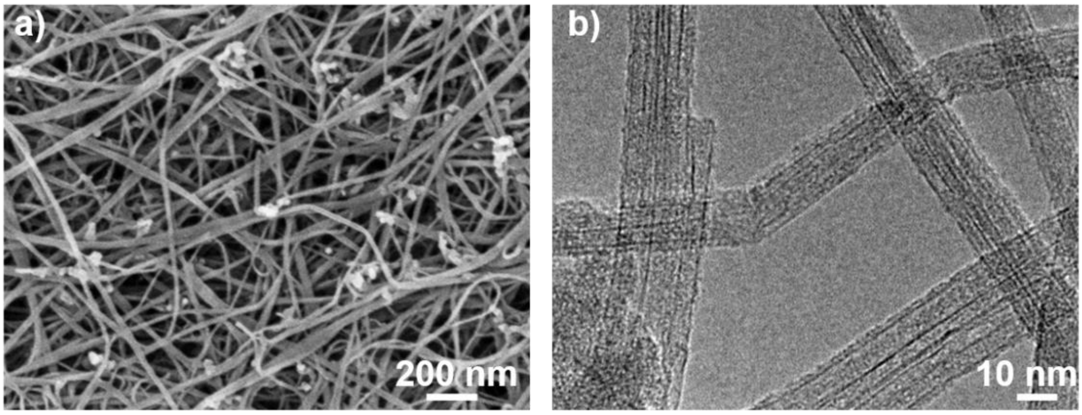

Figure S1. (a) SEM image of bare CNT film. (b) TEM image of bare CNTs.

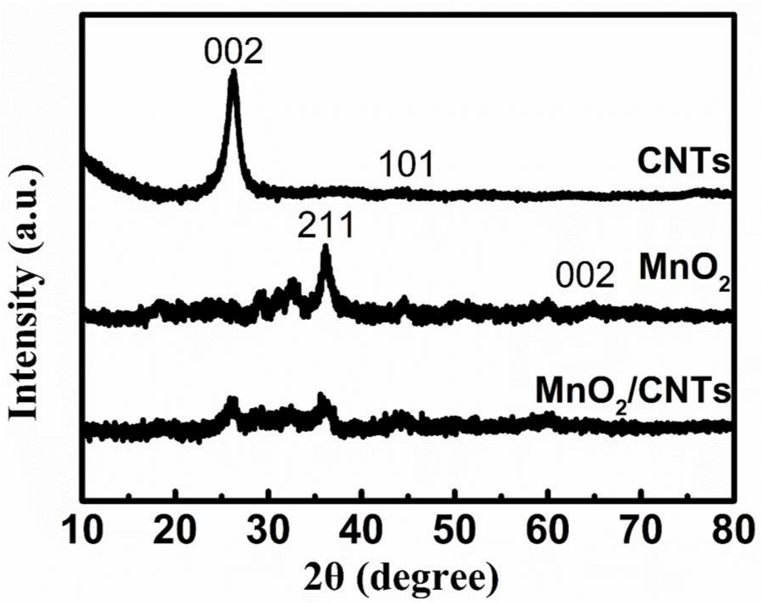

Figure S2. XRD patterns of bare CNT films, as-prepared MnO_2, and MnO_2/CNT composite film.

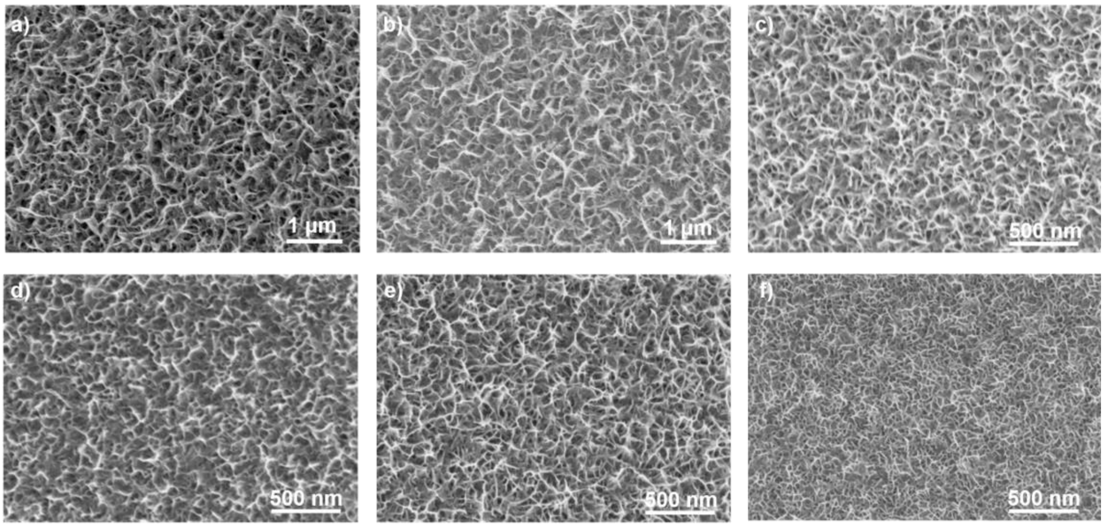

Figure S3. SEM images of MnO_2/CNT composites with different deposition time: (a) 10 min (b) 20 min (c) 25 min (d) 30 min (e) 35 min (f) 40 min.

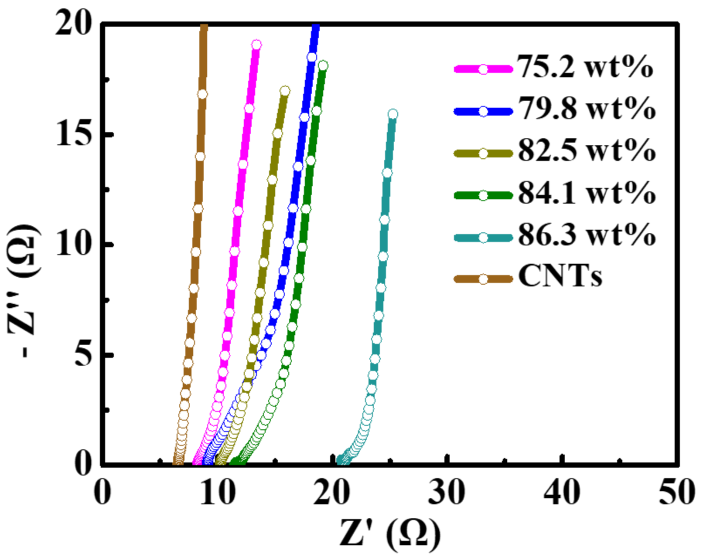

Figure S4. Nyquist plots of MnO_2/CNT composite electrodes with different mass loadings of MnO_2.

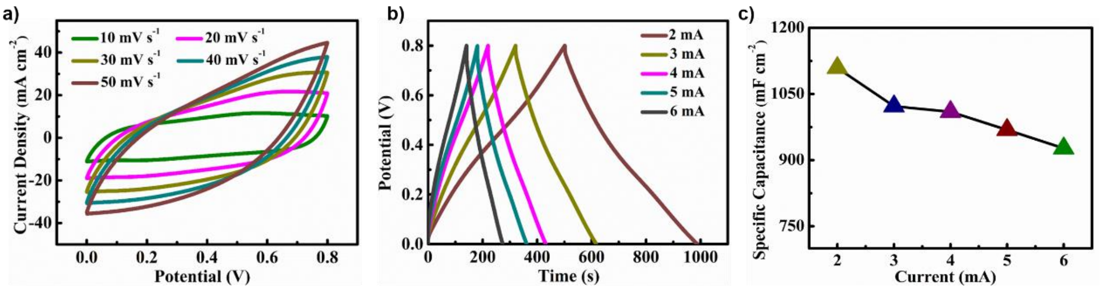

Figure S5. (a) CV curves of MnO_2/CNT composite electrodes at different scanning rates. (b) Charge-discharge curves of MnO_2/CNT composite electrodes at different current densities. (c) Areal specific capacitance of the MnO_2/CNT composite electrodes calculated from the GCD curves as a function of current density.

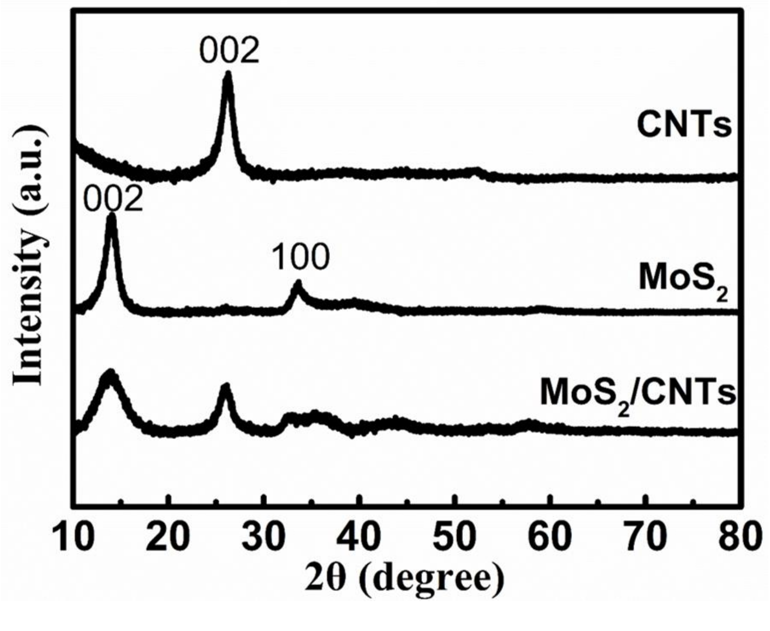

Figure S6. XRD patterns of bare CNT films, as-prepared MoS_2, and MoS_2/CNT composite film.

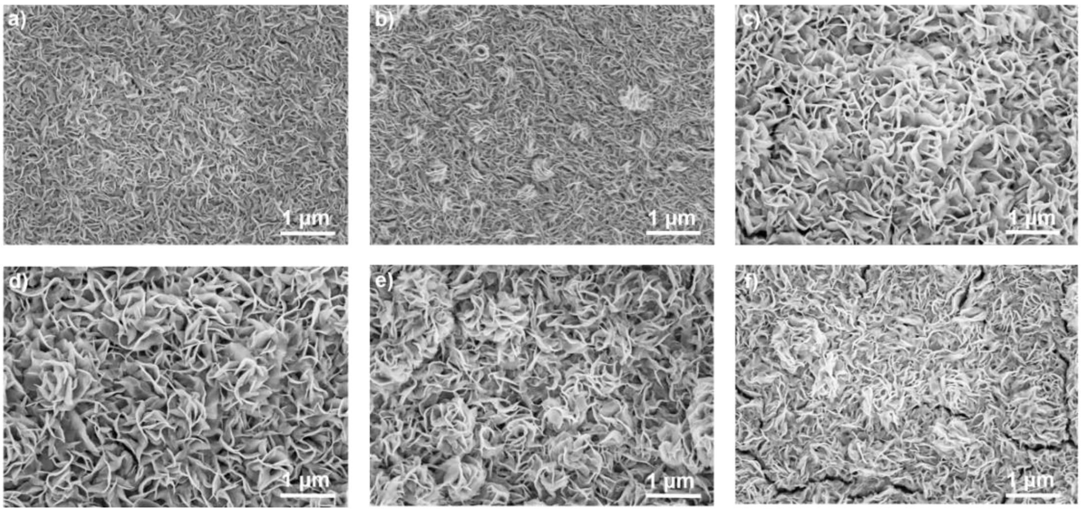

Figure S7. SEM images of MoS_2/CNT composites with different mass loadings of MoS_2: (a) 77.6 wt% (b) 82.2 wt% (c) 85.1 wt% (d) 86.2 wt% (e) 87.2 wt% (f) 89.0 wt%.

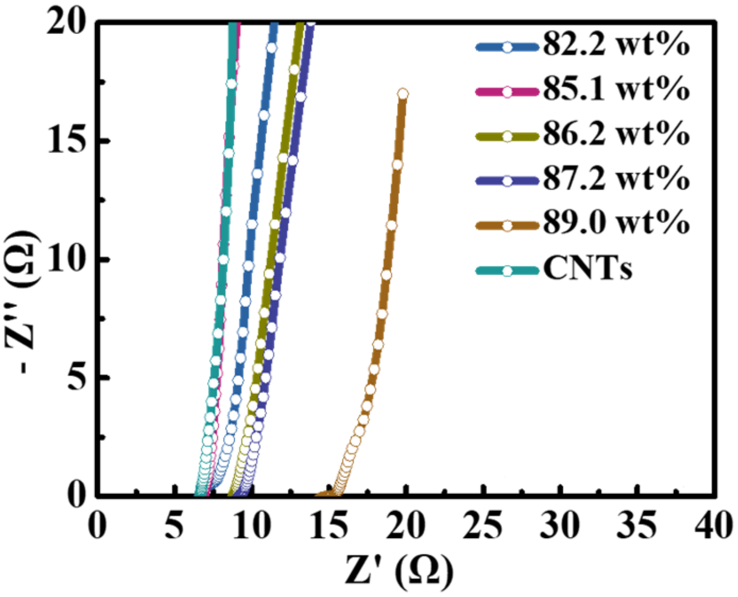

Figure S8. Nyquist plots of MoS_2/CNT composite electrodes with different mass loadings of MoS_2.

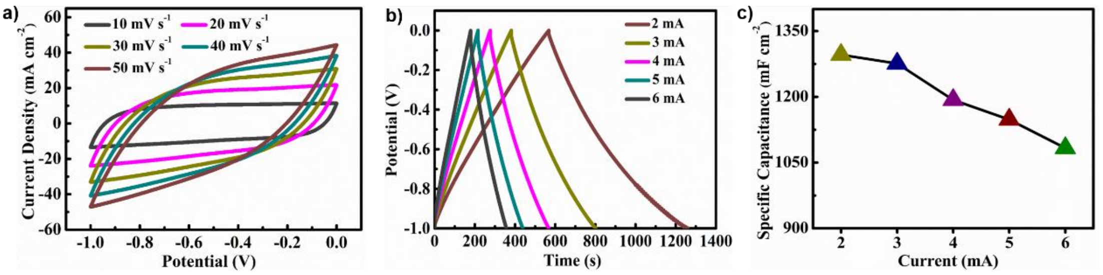

Figure S9. (a) CV curves of MoS_2/CNT composite electrodes at different scanning rates. (b) Charge-discharge curves of MoS_2/CNT composite electrodes at different current densities. (c) Areal specific capacitance of the MoS_2/CNT electrodes calculated from the GCD curves as a function of current density.

Table S1. Comparasion of performance of our ASC devices with other previous reports.

**Supercapacitor**	**Energy Density (mWh cm^{-3})**	**Power Density (mW cm^{-3})**	**Ref.**
MnO_2/CNT//MoS_2/CNT	**19.8**	195	**This work**
MnO_{2-x} NRs// RGO	0.25	1010	[1]
TiN@MnO_2 //EACC-10	1.5	1710	[2]
MnO_2@PEDOT:PSS@CNT//Microporous carbon@CNT	11.3	30	[3]
Co_9S_8 NRs//Co_3O_4@RuO_2	1.44	890	[4]
PANI@Au@PEN//PEDOT@Au@PEN	9	2.8	[5]
Ni@MnO_2@filter paper//Ni@AC@filter paper	0.78	2.5	[6]
MnO_2@rGO@SWCNT fiber//N-doped rGO@SWCNT fiber	5	929	[7]
CF@rGO@MnO_2//CF@thick rGO	1.23	270	[8]
CFT@polyaniline//Carbon-fiber thread (CFT)	2	11000	[9]
MnO_2@CF@CNPs//CF@CNPs	2.1	8000	[10]

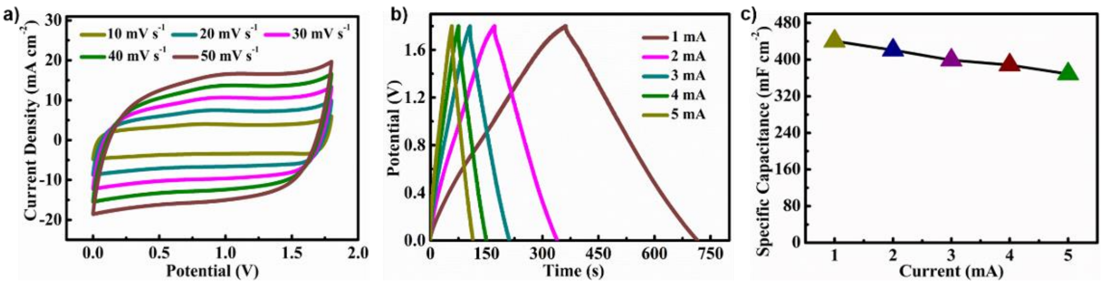

Figure S10. (a) CV curves of an ASC device measured at different scanning rates with a potential window of 1.8 V. (b) GCD curves of the ASC device at different current densities with a potential window of 1.8 V. (c) Areal specific capacitances calculated from the GCD curves as a function of the current density.

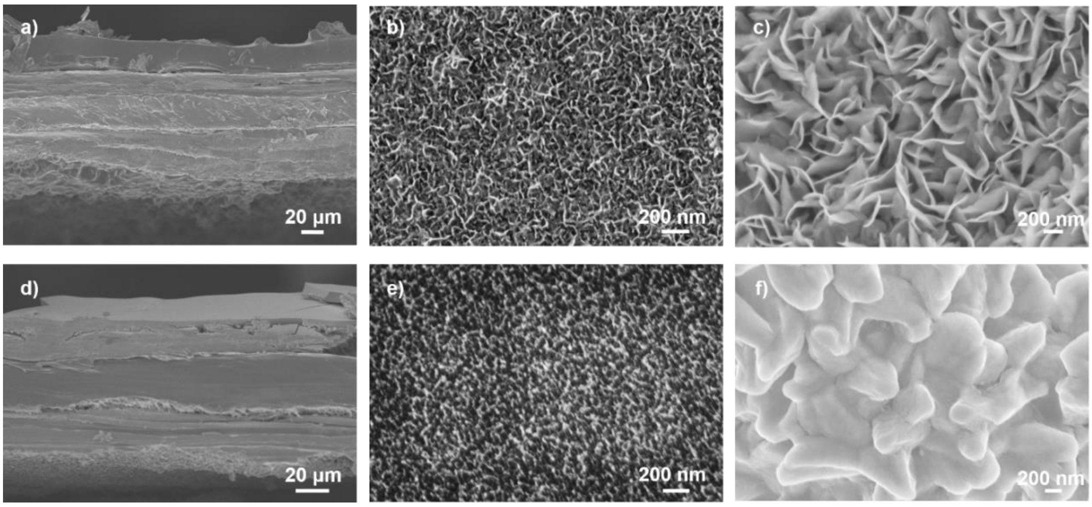

Figure S11. (a) Cross-sectional SEM image of ASC device before cycling charge-discharge test. (b,c) SEM images of MnO_2/CNT (b) and MoS_2/CNT (c) composites before cycling test. (d) Cross-sectional SEM image of ASC device after 2000 cycling charge-discharge tests. (e,f) SEM image of MnO_2/CNT (e) and MoS_2/CNT (f) composites composites after 2000 cycling charge-discharge tests.

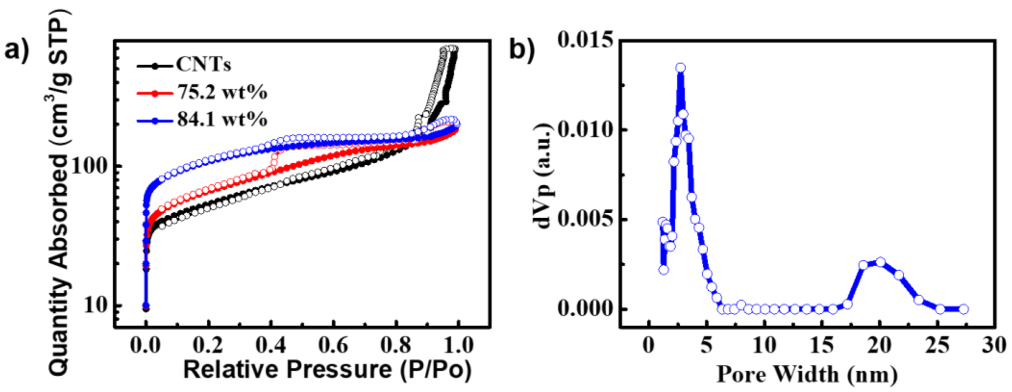

Figure S12. (a) N_2 adsorption-desorption isotherm at 77 K of bare CNT films, MnO_2/CNT composite electrodes with mass loadings of 75.2 wt% and 84.1 wt%. (b) Pore size distribution of MnO_2/CNT composite electrodes with mass loading of 84.1 wt%.

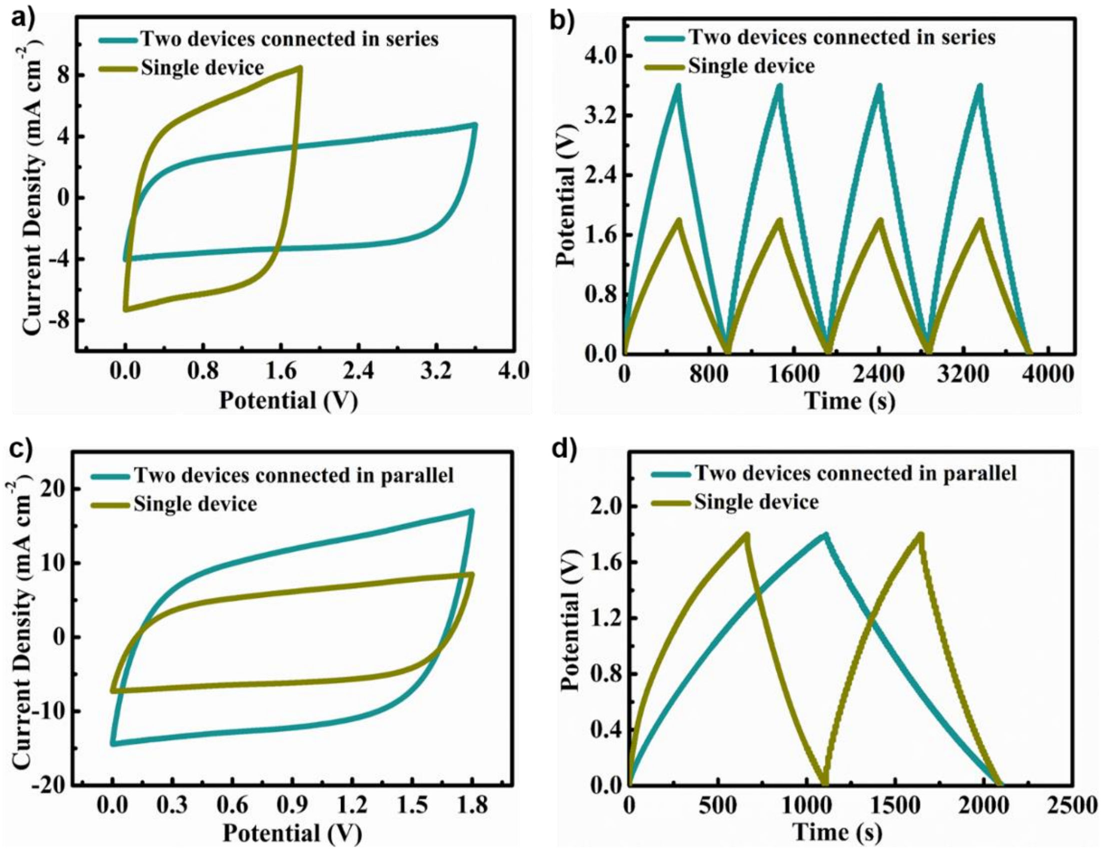

Figure S13. (a) CV curves of two ASC devices connected in series. (b) GCD curves of two ASC devices connected in series. (c) CV curves of two ASC devices connected in parallel. (d) GCD curves of two ASC devices connected in parallel.

References

[1] T. Zhai, S. Xie, M. Yu, P. Fang, C. Liang, X. Lu, Y. Tong, *Nano Energy* **2014**, 8, 255.

[2] W. Wang, W. Liu, Y. Zeng, Y. Han, M. Yu, X. Lu, Y. Tong, *Adv. Mater.* **2015**, 27, 3572

[3] X. Cheng, J. Zhang, J. Ren, N. Liu, P. Chen, Y. Zhang, J. Deng,Y. Wang, H. Peng, *J. Phys. Chem. C* **2016**, 120, 9685.

[4] J. Xu, Q. Wang, X. Wang, Q. Xiang, B. Liang, D. Chen, G. Shen, *ACS Nano* **2013**, 7, 5453.

[5] N. Kurra, R. Wang, H. N. Alshareef, *J. Mater. Chem. A* **2015**, 3,7368.

[6] L. Zhang, P. Zhu, F. Zhou, W. Zeng, H. Su, G. Li, J. Gao, R. Sun, C. Wong, *ACS Nano* **2016**, 10, 1273.

[7] D. Yu, K. Goh, Q. Zhang, L. Wei, H. Wang, W. Jiang, Y. Chen, *Adv.Mater.* **2014**, 26, 6790.

[8] Z. Zhang, F. Xiao, J. Xiao, S. Wang, *J. Mater. Chem. A* **2015**, 3,11817.

[9] H. Jin, L. Zhou, C. L. Mak, H. Huang, W. M. Tang, H. L. W. Chan, *Nano Energy* **2015**, 11, 662.

[10] H. Jin, L. Zhou, C. L. Mak, H. Huang, W. M. Tang, H. L. Wa Chan, *J. Mater. Chem. A* **2015**, 3, 15633.
